# Novel expression of *Haemonchus contortus* vaccine candidate aminopeptidase H11 using the free-living nematode *Caenorhabditis elegans*

**DOI:** 10.1186/1297-9716-44-111

**Published:** 2013-12-01

**Authors:** Brett Roberts, Aristotelis Antonopoulos, Stuart M Haslam, Alison J Dicker, Tom N McNeilly, Stephanie L Johnston, Anne Dell, David P Knox, Collette Britton

**Affiliations:** 1 Institute of Infection, Immunity and Inflammation, College of Medical, Veterinary and Life Sciences, University of Glasgow, Bearsden Road, Glasgow G61 1QH, UK; 2 Department of Life Sciences, Imperial College London, South Kensington Campus, London SW7 2AZ, UK; 3 Division of Parasitology, Moredun Research Institute, Pentlands Science Park, Penicuik, Edinburgh EH26 0PZ, UK

## Abstract

With the problem of parasitic nematode drug resistance increasing, vaccine development offers an alternative sustainable control approach. For some parasitic nematodes, native extracts enriched for specific proteins are highly protective. However, recombinant forms of these proteins have failed to replicate this protection. This is thought to be due to differences in glycosylation and/or conformation between native and recombinant proteins. We have exploited the free-living nematode *Caenorhabditis elegans* to examine its suitability as an alternative system for recombinant expression of parasitic nematode vaccine candidates. We focussed on *Haemonchus contortus* aminopeptidase H11 glycoprotein, which is enriched in a gut membrane fraction capable of inducing significant protection against this important ovine gastrointestinal nematode. We show that *H. contortus* H11 expressed in *C. elegans* is enzymatically active and MALDI mass spectrometry identifies similar di- and tri-fucosylated structures to those on native H11, with fucose at the 3- and/or 6-positions of the proximal GlcNAc. Some glycan structural differences were observed, such as lack of LDNF. Serum antibody to native H11 binds to *C. elegans* recombinant H11 and most of the antibody to rH11 or native H11 is directed to glycan moieties. Despite these similarities, no reduction in worm burden or faecal egg count was observed following immunisation of sheep with *C. elegans*-expressed recombinant H11 protein. The findings suggest that the di- and tri-fucosylated N-glycans expressed on rH11 do not contribute to the protective effect of H11 and that additional components present in native H11-enriched extract are likely required for enhancing the antibody response necessary for protection.

## Introduction

Parasitic nematode infections of livestock are responsible for significant economic losses and welfare concerns globally. Control currently relies on the use of anthelmintic drugs, however the widespread problem of parasite anthelmintic resistance means that this approach is becoming unsustainable [1,2]. The recent introduction of a new class of anthelmintic, the aminoacetonitrile derivatives (ADDs; monepantel), offers an alternative [3]. However resistance to this new drug class has already been reported in nematodes of sheep and goats in less than 2 years of use [4]. Alternative, sustainable control measures, such as vaccination and improved pasture management, are urgently needed. Significant protection against livestock nematode infections has been achieved following vaccination with native protein extracts, demonstrating that vaccination is feasible [5-7]. However, from a production and cost perspective, the long term aim is to develop molecularly defined vaccines amenable to commercial development, without the need for native parasite material and the associated ethical and safety concerns. Currently there is no molecularly defined vaccine available for any parasitic nematode.

A significant amount of data is available from vaccine trials against the blood-feeding gastrointestinal (GI) nematode of sheep and goats, *Haemonchus contortus.* Native proteins extracted from the adult parasite gut or from excretory-secretory (ES) products are capable of inducing high levels of protection (up to 90% reduction in faecal egg counts (FEC) and 75% reduction in worm burden) [7]. Protective gut fractions include a galactose-binding glycoprotein complex termed H-gal-GP enriched for metallo and aspartic proteases, a thiol-binding fraction enriched for cysteine proteases, and a Concanavalin A binding fraction enriched for aminopeptidase H11. However, attempts to mimic the protective effects of these native extracts using recombinant forms of the enriched proteases expressed in bacteria, yeast or insect cells have proved unsuccessful [6,8]. Protection studies against the cattle GI nematode *Ostertagia ostertagi* have similarly demonstrated significant reductions in egg output using an ES fraction highly enriched for two activation-associated secreted proteins (ASP-1 and ASP-2) [9]. However, vaccination with baculovirus-expressed ASP-1 protein failed to induce any protection [10]. There has been much speculation as to why recombinant parasitic nematode proteins fail to induce protective immunity. Possible explanations include incorrect folding, lack of glycosylation of bacterially-expressed proteins, inappropriate glycosylation of yeast or insect-expressed proteins, induction of lower avidity antibodies or, alternatively, that the dominant proteins identified in protective native fractions are not solely responsible for protection [8].

Gene rescue studies have previously demonstrated that parasite proteins can be expressed in a biologically active form in the free-living nematode *C. elegans*[11-14]. However, expression of parasite proteins with complex post-translational modifications has not been tested in *C. elegans*. In this study we focus on *H. contortus* H11 aminopeptidase, due to the high level of protection achieved with native gut extracts enriched for H11 and the unusual glycosylation identified on the native protein. Previous mass spectrometric analysis identified unusual fucosylated modifications on native H11, including core α1-3 and α1-6 fucosylation [15]. The former is not found on mammalian glycans and has been shown to be highly antigenic when present on plant and insect glycoproteins [16]. The core α1-3 Fuc epitope is recognised by IgE antibody from *H. contortus* infected sheep and is speculated to contribute to the induction of a Th2 response [17]. Core α1-3 and α1-6 fucosylation structures have also been identified on *C. elegans* glycoproteins (though it should be noted that these are often additionally substituted with galactose residues) [18-20] and have been shown to induce Th2 type immune responses in mice, similar to parasite glycans [21]. α1-3 and α1-6 fucosyltransferases have been characterised from *C. elegans*[22], indicating that glycosylation pathways and the resulting glycan modifications are similar in free-living and parasitic nematodes. *C. elegans* may therefore present a suitable system for expression and analysis of parasite glycans as important immunogens. Here we express recombinant *H. contortus* H11 protein in *C. elegans* and characterise the glycosylation pattern, enzymatic activity and antibody recognition of native and recombinant protein. Our findings have important relevance to expression of other nematode vaccine candidates and requirements for protective immunity.

## Materials and methods

### Identification of genomic location of *H. contortus* H11 genes

The available *H. contortus* genome data (strain MHco3(ISE)) [23,24] was searched by tBLASTn using amino acid sequences of all available H11 isoforms. This identified a number of overlapping scaffolds encoding known H11 sequences and identified a novel sequence, named H11-5 [GenBank Accession number KF381362]. Tandem arrangement of H11 genes was indicated by scaffold sequence analysis and confirmed experimentally by PCR on genomic DNA, extracted by standard method from *H. contortus* adult worms ((MHco3(ISE) strain), using DNA primers designed to the 5′ and 3′ ends of each H11 gene coding sequence. All primer sequences are available on request.

### RNA extraction from *H. contortus* and semi-quantitative RT-PCR

Total RNA was extracted from *H. contortus* adult worms using an RNAeasy kit (Qiagen) after grinding the worms in liquid nitrogen. Reverse Transcription was performed using an AffinityScript cDNA Synthesis kit (Stratagene) as per manufacturer’s instructions. Semi-quantitative RT-PCR was carried out as described previously [25], using constitutively expressed superoxide dismutase gene (*Hc-sod-1*) as a reference [26]. Relative levels of each H11 gene were determined using ImageJ [27].

### Generation of *H. contortus* H11 expression constructs

An expression cassette containing 1.76 kb of promoter sequence of *C. elegans* cathepsin L protease gene *cpl-1* and 500 bp of *Ce-cpl-1* 3′ UTR was generated in the TOPO 2.1 vector (Invitrogen) by standard cloning methods as previously described [12]. The cDNA region encoding the putative signal peptide sequence (first 29 amino acids) of the *H. contortus Hmcp-6* gene (accession number GQ223792) was inserted between the cloned *Ce-cpl-1* promoter and 3′ UTR regions. The *H. contortus H11* coding sequence for each isoform (approximately 2.9 kb) was inserted into the expression cassette between the *Hmcp-6* signal peptide-encoding region and *Ce-cpl-1* 3′UTR using *Nru* I and *Xho* I restriction sites at these positions. The *H11* coding regions were amplified using *PfuUltra* II Fusion HS DNA Polymerase (Stratagene) and *H. contortus* adult worm cDNA as template. The 5′ primers were designed to amplify downstream of the encoded N-terminal transmembrane domain (35 amino acids) of each *H11* gene. The 3′ primers contained *Afe* I sites to allow for the insertion of a 10 amino acid C-terminal His tag-encoding sequence. A *C. elegans* synthetic intron (SI) was inserted into available blunt end restriction sites within the *H11* genes to improve transgene expression [28]. A diagrammatic representation of the final H11 expression construct is shown in Additional file 1. The integrity of all constructs was verified by automated DNA sequencing (Eurofins MWG).

### *C. elegans* transgenesis

Each *H11* gene expression construct was microinjected into the gonads of *C. elegans* adult hermaphrodite worms of strain DR96 (*unc-76(e911)*) as simple free arrays at a final concentration of 10 μg/mL, together with the rescue marker plasmid p76-16B [29] at a final concentration of 10 μg/mL and 1 kb ladder DNA (100 μg/mL) [30]. Transgenic animals were selected on the basis of reversion from an uncoordinated (*unc*) to wild-type phenotype.

### Culture of *C. elegans* transgenic worms and protein purification

*C. elegans* worms transformed with the *H11* expression constructs were grown on 9 cm diameter peptone rich plates seeded with N22 bacteria [31] for approximately seven days. Worms were washed from the plates in 0.1 M NaCl and separated from bacteria by centrifugation at 3000 rpm on a 60% (w/v) sucrose gradient. Worms were collected from the upper phase and washed three times in ice cold 0.1 M NaCl by centrifugation at 3000 rpm. The worm pellet was resuspended in an equal volume of 1× lysis buffer (50 mM NaPO_4_, 300 mM NaCl, 10 mM imidazole) and stored at -70 °C. After thawing on ice, the volume was made up to 10 mL with 1× lysis buffer. Worms were sonicated for 10 cycles of 10 s on/20 s off on ice, followed by homogenisation in a standard glass homogeniser. The worm extract was centrifuged at 13 000 rpm for 3 min and the cleared lysate collected and stored on ice. The worm pellet was resuspended in 3 mL of 1× lysis buffer and re-sonicated and homogenised. After centrifugation, the lysates were pooled, added to 300 μL of HisPur Cobalt Resin (ThermoScientific) and mixed gently on a roller at 4 °C for 2 h. The solution was loaded onto a 1 mL polypropylene column (Qiagen) and the resin washed with 4 mL lysis buffer, followed by 4 mL of lysis buffer containing 0.04 M imidazole. Bound protein was eluted with six aliquots of 150 μL of 1× lysis buffer containing 0.125 M imidazole. Eluted protein was dialysed against 1 × PBS using Slide-a-Lyzer cassettes (Thermo Scientific) and the protein concentration estimated using the Coomassie Protein Assay Reagent (Thermo Scientific). Protein was analysed by 4-15% gradient SDS polyacrylamide gel electrophoresis (SDS-PAGE; Bio-Rad minisystem) followed by Coomassie staining (0.25%).

### Protein identification by mass spectrometry

A sample of *C. elegans*-expressed recombinant H11-4 (rH11-4) protein purified by cobalt chelation chromatography was subjected to SDS-PAGE, stained with Coomassie blue and the major band excised. Standard mass spectrometry procedure was performed with in-gel trypsin digest, extraction of tryptic peptides, fractionation by RP-HPLC and online nano-electropsray tandem mass spectrometry (MS). Data was searched against the National Center for Biotechnology Information (NCBI) database using the Mascot search engine (Proteomics Services, University of Glasgow). Native H11 protein was extracted from *H. contortus* adult worms (day 28) by solubilisation of a PBS homogenate pellet in 1% thesit and purified using Concanavalin A (Con A) sepharose (Sigma). Protein extract was separated on a 4-15% SDS-PAGE gel, stained with Coomassie blue and the major band of approximately 110 kDa excised and subject to MS as described above (Proteomics Unit, Moredun Research Institute).

### SDS-PAGE and Western blotting

Protein samples were boiled for 5 min in an equal volume of 2 × SDS-PAGE loading buffer containing 5% β-mercaptoethanol and separated by 4-15% gradient SDS-PAGE. Proteins were visualised with Coomassie blue or western blotting carried out by transferring the proteins onto PVDF membrane (Amersham). Blots were probed with a variety of antibodies: mouse anti-HIS (C-terminal) (Invitrogen; 1 in 4000 dilution), sheep antiserum to native H11-enriched gut membrane extract (1 in 2000 dilution), Con A-horseradish peroxidase (HRP; Sigma L6397; 0.5 μg/mL), mouse IgA TEPC-15 antibody (Sigma M1421, 1 in 2500 dilution) and sheep antiserum raised to *C. elegans*-expressed recombinant H11 (1 in 2000 dilution). HRP-conjugated secondary antibodies were routinely used at 1 in 10 000 dilution and binding detected using the Enhanced Chemiluminscent (ECL) system (GE Healthcare).

### Site-directed mutagenesis of *H. contortus* H11-4

The three putative N-glycosylation sites of H11-4 (identified as described in [32]) were altered by site-directed mutagenesis using the QuikChange Lightning Multi Site-Directed Mutagenesis Kit (Stratagene) according to the manufacturer’s instructions. Three gene specific primers were used in which the codon for asparagine was altered to that for glutamine. DNA sequencing confirmed alteration of all three codons (Eurofins MWG).

### Mild periodate treatment of H11 proteins

PVDF membrane containing native H11-enriched extract or rH11-4 protein was washed with 30% isopropanol, 10% acetic acid for 5 min, before being rinsed in 50 mM sodium acetate pH 4.5, 50 mM NaCl buffer. The following incubations were all performed at 4 °C in the dark. The blot was incubated in the above buffer containing 10 mM sodium metaperiodate for 1 h, rinsed 3 times in water before being incubated in 20 mM Tris, pH 7.2, 150 mM NaCl, 200 mM glycerol for 20 min. After rinsing 3 times in 20 mM Tris, pH 7.2, 150 mM NaCl, the standard immunoblotting protocol was followed [33].

### Glycan analysis

A sample of rH11-4 protein was processed as previously described [34]. Briefly, rH11-4 protein was subjected to reduction in 4 M guanidine HCl (Pierce) containing 2 mg/mL dithiothreitol, carboxymethylation and trypsin digestion, and the digested glycoprotein was purified by C_18_-Sep-Pak (Waters Corp., Hertfordshire, UK). N-linked glycans were released by peptide:N-glycosidase F (EC 3.5.1.52, Roche Applied Science) and peptide:N-glycosidase A (EC 3.5.1.52, Roche Applied Science) digestions. N-linked glycans were then permethylated using the sodium hydroxide procedure, and finally, the permethylated N-linked glycans were purified by C_18_-Sep-Pak. All permethylated samples were dissolved in 10 μL of methanol, and 1 μL of dissolved sample was premixed with 1 μL of matrix (20 mg/mL 2,5-dihydroxybenzoic acid in 70% (v/v) aqueous methanol). The mixture was then spotted onto a target plate (2 × 0.5 μL), and dried under vacuum. MS data were acquired using a Voyager-DE STR MALDI-TOF (Applied Biosystems, Darmstadt, Germany). MS/MS data were acquired using a 4800 MALDI-TOF/TOF (Applied Biosystems) mass spectrometer. The collision energy was set to either 1 or 2 kV, and argon was used as collision gas. The 4700 calibration standard kit, Calmix (Applied Biosystems), was used as the external calibrant for the MS mode of both instruments, and [Glu1] fibrinopeptide B human (Sigma) was used as an external calibrant for the MS/MS mode of the MALDI-TOF/TOF instrument.

The MS and MS/MS data were processed using Data Explorer 4.9 Software (Applied Biosystems). The spectra were subjected to manual assignment and annotation with the aid of the glycobioinformatics tool, GlycoWorkBench [35]. The proposed assignments for the selected peaks were based on ^12^C isotopic composition together with knowledge of the biosynthetic pathways. The proposed structures were then confirmed by data obtained from MS/MS experiments.

### Aminopeptidase activity assays

Aminopeptidase activity was determined by standard assay using l-leucine p-nitroanilide substrate (l-Leu-pNA; Sigma). Protein sample (500 ng/well) was mixed with 100 μL 50 mM NaPO_4_ buffer (pH5.5-7.5) and 10 μL l-Leu-pNA (0.5 mM final concentration). The initial and final absorbance (after 2 h at 37 °C and overnight incubation at room temperature) was determined at 405 nm using a Beckman DU530 spectrophotometer. To test inhibitor sensitivity, assays were carried out as above, but with pre-incubation for 10 min in amastatin or bestatin (both at 10 μM final concentration) prior to the addition of pNA substrate.

### Vaccine trial

Six month old indoor housed, worm-free Suffolk-cross lambs were allocated into groups of seven, balanced for sex and weight. One group received three immunisations (sub-cutaneously) of *C. elegans*-expressed rH11-1 and rH11-4 protein (a combination of 10 μg of each protein) or rH11-4 and rH11-5 co-expressed in *C. elegans* (20 μg total protein) at three weekly intervals and were challenged with 5000 infective L3 stage *H. contortus* on the day of the final immunisation (day 42). Vax Saponin (Guiness Chemical Products Ltd) was used as adjuvant at a final concentration of 1 mg/mL and the final vaccine volume was adjusted to 1 mL with 1× TBS. Blood was sampled at weekly intervals and FEC monitored three times weekly from day 14 after challenge until necropsy at day 35 when worm numbers were counted, as previously described [36]. Control groups were injected with 1 × TBS and adjuvant only or with 40 μg of native gut membrane extract enriched for H11 plus adjuvant, following the method of [37]. All experimental procedures were approved by the Moredun Research Institute Experiments and Ethics committee and carried out in accordance with the Animals (Scientific Procedures) Act of 1986.

### ELISA

Microtitre plates were coated overnight at 4 °C with 1 μg/mL of a combination of rH11-1 and rH11-4 proteins or rH11-4 and rH11-5 co-expressed proteins diluted in 50 mM bicarbonate buffer, pH 9.6. The avidity of serum antibody from lambs vaccinated with native H11-enriched extract or rH11 protein against the homologous protein was estimated using the thiocyanate elution method [38], with the addition of 0-5 M KSCN to all washes. Avidity was estimated as the concentration of KSCN which resulted in a 50% reduction in OD value at a particular serum dilution (1/800). Mild sodium periodate-treatment (final concentration 5 mM) was performed after coating of protein to wells, as previously described [39].

Antibody isotype reactivity was determined using standard ELISA method with primary antibody at 1/50 dilution and probing with mouse anti-ovine IgG (clone GT-34, Sigma-Aldrich; 1/2500 dilution), IgM (clone VPM13, Moredun Research Institute; 1/1000 dilution), IgE (clone 2 F1, Moredun Research Institute; 1/100 dilution) or IgA (AbD Serotec clone K84 2 F9; 1/1000 dilution). Secondary antibody binding was detected with anti-mouse IgG-HRP (Sigma-Aldrich A2304, 1/1000).

### Statistical analysis

Statistical analysis of the FEC and worm burden results was carried out following the guidelines set out in [40] and data analysed using Excel 2010.

## Results

### Developmental expression of H11 gene family

**Figure 1:**
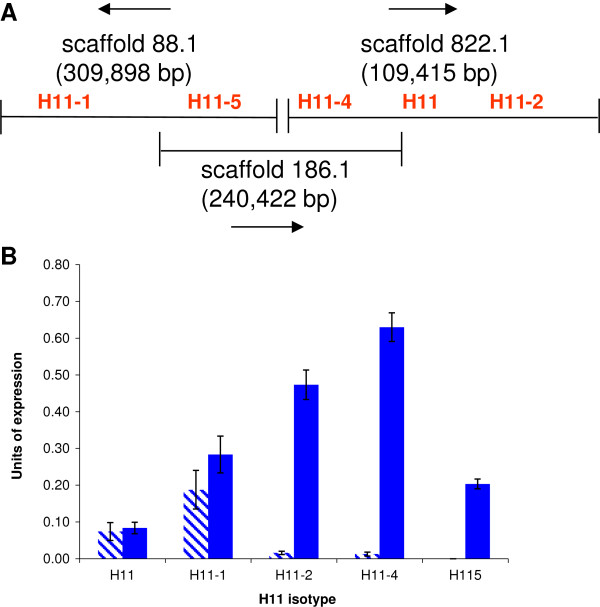
**Genomic organisation and developmental expression of *H. contortus *H11 isoforms. (A)***H. contortus* overlapping genomic scaffold sequences indicating tandem arrangement of all five H11 isoforms. Arrows indicate the direction of transcription from each scaffold. **(B)** Semi-quantitative RT-PCR of the five *H. contortus* H11 genes in infective L3 larvae (striped bars) and in adult worms (blue bars). Expression of each gene is shown relative to constitutively expressed control gene *Hc-sod-1.* Mean of triplicate PCRs is shown, with error bars showing the standard error of the mean.

Genes encoding four different isoforms of *H. contortus* H11 were previously identified (H11, H11-1, H11-2 and H11-4) and in this study we identified a fifth isoform (H11-5) from the available *H. contortus* genome data. Analysis of overlapping *H. contortus* scaffolds, in addition to performing PCR on *H. contortus* genomic DNA, showed that all 5 isoforms are tandemly arranged in the genome (Figure 1A). Our findings expand on previous data [41], which showed linkage of H11-4 and H11 genes, and suggest that the H11 gene family has arisen through recent duplication and divergence. The encoded proteins share 62-75% amino acid identity, with the zinc binding domain (HELAH) and exopeptidase motif (GAMEN) characteristic of N-aminopeptidases highly conserved in all isoforms (Additional file 2). All H11 proteins contain predicted N-glycosylation sites, as shown in the amino acid sequence alignment in Additional file 1.

Semi-quantitative RT-PCR identified transcript for all five isoforms in adult worms, with significantly lower expression in infective L3 stage larvae (Figure 1B). Different isoforms showed different patterns of expression, with H11-1 the most abundant isoform in infective L3 larvae, H11-4 the most abundant isoform in adult worms and H11-5 only expressed in adult female worms (not shown). Subsequent RNA seq data [23] confirmed the male-specific expression of H11-5 and identified significant enrichment of transcripts encoding H11-4, H11 and H11-2 in *H. contortus* adult gut tissue. All five H11 isoforms are present in native H11 protein extract, as indicated by MS peptide sequencing, with more peptide matches found to H11-1 than to the other isoforms (Mascott scores ranged from 390–5567). Based on this data, H11-1, H11-4 and H11-5 were selected for large-scale protein expression in *C. elegans,* followed by glycan and enzymatic characterisation, and vaccine testing.

### Recombinant expression of *H. contortus* H11 proteins using *C. elegans*

**Figure 2:**
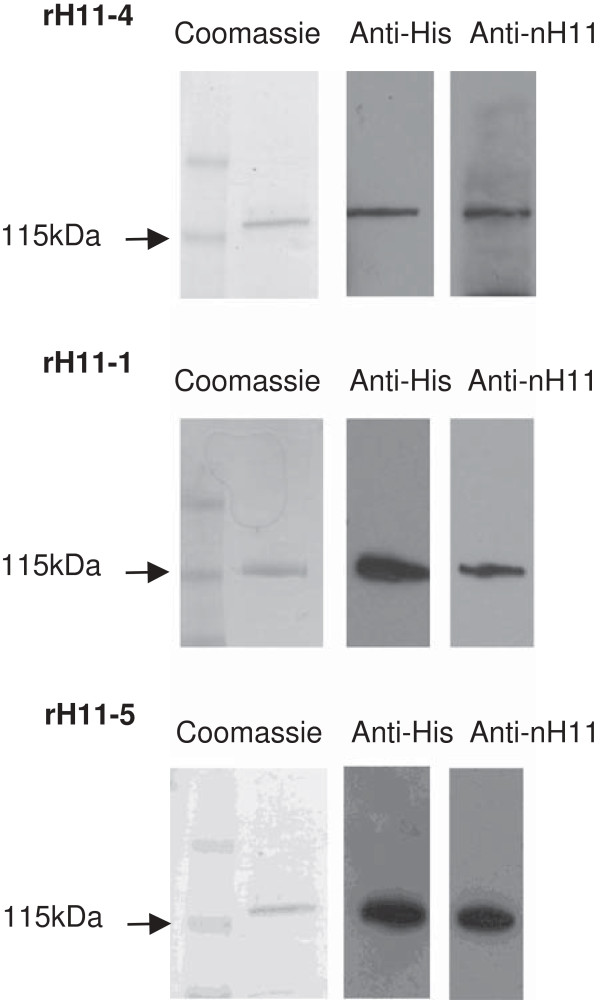
**Expression and purification of recombinant H11-4, H11-1 and H11-5 expressed in *C. elegans*.** rH11 proteins purified by cobalt chelation chromatography were separated by 4-15% SDS-PAGE and stained with Coomassie blue or transferred to PVDF membrane and probed with anti-His (C-terminal) antibody or antiserum to native H11-enriched extract (Anti-nH11). Antiserum from animals prior to H11-extract immunisation showed no reactivity to rH11 isoforms.

*H. contortus* H11 isoforms were expressed in transgenic *C. elegans* as soluble proteins by substituting the N-terminal transmembrane domain with a short signal peptide sequence. Previous studies demonstrated that a soluble form of native H11, in which the transmembrane domain was removed, was equally protective compared to full-length H11 [8]. Coomassie staining and Western blotting using antibody to the C-terminal His tag or antiserum to native H11-enriched extract (nH11) confirmed that recombinant H11 protein (rH11) was the major cobalt-binding protein in a PBS-soluble extract of *C. elegans* mixed stage transgenic worms (Figure 2). Pre-bleed serum prior to H11-extract immunisation showed no reactivity. The identity of rH11 was also confirmed by MS peptide sequencing. While characterisation focussed on H11-1, H11-4 and H11-5, all 5 isoforms were able to be expressed in recombinant form in *C. elegans* and antiserum raised to native H11-enriched extract recognised all rH11 isoforms. For each rH11 protein, the yield was approximately 70–100 μg purified protein per mL packed volume of *C. elegans* transgenic worms.

### Enzymatic activity of recombinant H11 proteins expressed in *C. elegans*

**Figure 3:**
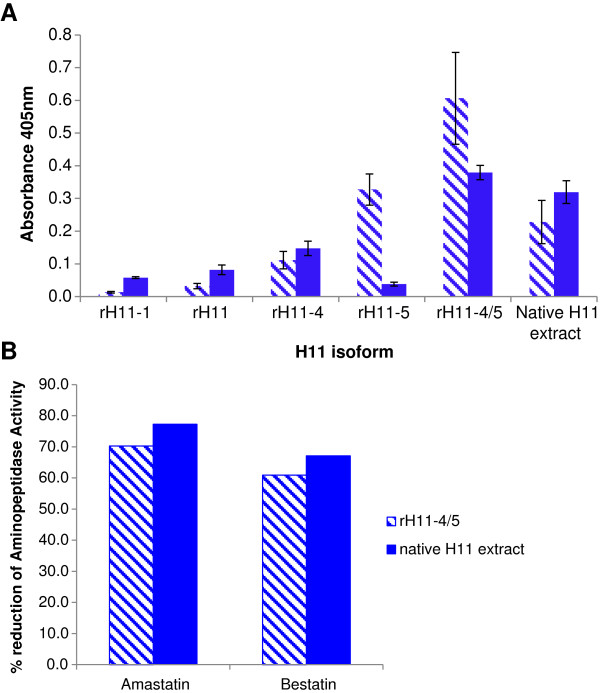
**Aminopeptidase activity of *C. elegans*-expressed recombinant H11 proteins. (A)** Aminopeptidase activity of rH11 proteins was measured as the change in absorbance at 405 nm using l-leucine p-nitroanilide as substrate. Activity was measured at pH5.5 (striped bars) and pH7 (blue bars) for singly expressed rH11 proteins, rH11-4 and rH11-5 co-expressed in *C. elegans* (rH11-4/5) and native H11-enriched extract. 500 ng total protein was added per assay. The results of triplicate assays are shown with error bars representing the standard error. **(B)** Reduction in aminopeptidase (AP) activity of rH11-4/5 co-expressed proteins (striped bars) and native H11 extract (blue bars) was measured in the presence of amastatin or bestatin, both at 10 μM final concentration. Assays were carried out in duplicate and % reduction calculated relative to pre-incubation in buffer alone.

Aminopeptidase activity of rH11 proteins expressed in *C. elegans* was examined by standard aminopeptidase enzyme assay using l-leucine p-nitroanilide (pNA) as substrate. H11 recombinant proteins were enzymatically active, with rH11-4 and rH11-5 showing greater activity than H11 and H11-1 isoforms (Figure 3). rH11-4 and rH11-5 proteins co-expressed and purified from the same *C. elegans* transgenic line (rH11-4/5) showed an increase in aminopeptidase activity relative to singly-expressed forms, which was consistent in all assays, and suggests possible interaction and/or stablisation of these isoforms (Figure 3A). H11-5 showed greater activity at pH 5.5, while H11-4 was more active at pH 7.0, similar to native H11-enriched extract. Aminopeptidase activity of the rH11-4 and rH11-5 co-expressed proteins and native H11-enriched extract was inhibited to a similar degree by standard aminopeptidase inhibitors amastatin and bestatin (60-70% reduction; Figure 3B). The activity and inhibition observed suggested that the recombinant H11 proteins were folded appropriately into active enzymes.

### *C. elegans*-expressed rH11 protein is N-glycosylated

**Figure 4:**
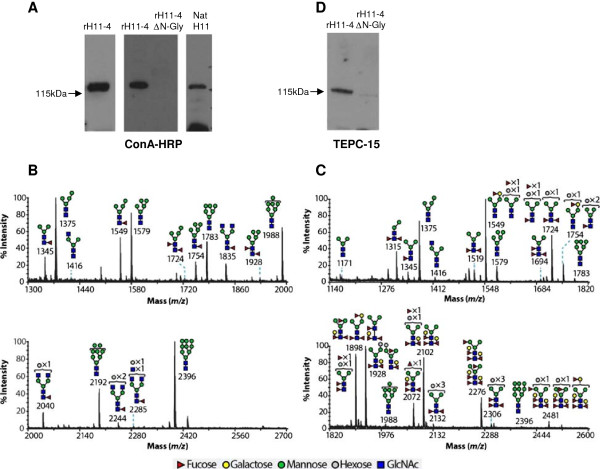
***C. elegans*-expressed rH11-4 protein is modified by N-linked glycosylation and phosphorylcholine. (A)** Purified rH11-4 protein, rH11-4 protein in which the three potential N-glycosylation sites were mutated by site directed mutagenesis (rH11-4 ΔN-Gly) and native H11-enriched extract were separated by 4-15% SDS-PAGE, transferred to PVDF membrane and probed with HRP-labelled Con A lectin. **(B and C)** MALDI-TOF mass spectrometry (MS) profiles of N-linked glycans from rH11-4 protein. N-glycans of purified rH11-4 were released from tryptic glycopeptides by digestion with PNGase F **(B)** or PNGase A **(C)**. Profiles of *N*-glycans are from the 50% MeCN fraction from a C_18_ Sep-Pak (Materials and Methods). All molecular ions are [M + Na]^+^. Putative structures are based on composition, tandem MS and biosynthetic knowledge. Structures that show sugars outside of a bracket have not been unequivocally defined. **(D)** Western blot of rH11-4 and rH11-4 ΔN-Gly probed with phosphorylcholine (PC) antibody TEPC-15.

Native *H. contortus* H11 protein binds to Concanavalin A lectin (Con A) [42] and previous mass-spectrometric analysis identified N-glycans with highly fucosylated core structures [15]. Interaction with Con A-HRP was examined by Western blotting, and showed strong binding to native H11-enriched extract and to recombinant H11-4 (Figure 4A), rH11-1 and rH11-5 proteins. No ConA-HRP binding was observed with protein expressed from a rH11-4 gene construct in which the three potential N-glycosylation sites had been altered by site-directed mutagenesis (H11-4ΔN-Gly), indicating specific interaction with N-linked glycans (Figure 4A).

To identify the specific N-linked glycan structures present on *C. elegans*-expressed rH11-4 protein, detailed mass-spectrometric analysis was carried out after PNGase F and PNGase A enzymatic release. N-glycans were analyzed by matrix-assisted laser desorption/ionization–time-of-flight (MALDI-TOF) mass spectrometry (MS), as well as by collisionally activated dissociation (CAD) on a MALDI-TOF-TOF instrument [tandem mass spectrometry (MS/MS)]. Mixtures of N-glycans were permethylated before MS and MS/MS analyses. The spectrum of PNGase F released glycans was dominated by high mannose structures (*m/z* 1579–2396 Man5-9GlcNAc2) (Figure 4B). This is fully consistent with the positive Con A binding experiments. In addition pauci-mannose type (*m/z* 1345–1928 Fuc0-2Hex3-5GlcNAc2) and minor amounts of complex type glycans (*m/z* 1416–2285 Fuc0-1Hex3-5GlcNAc2-4) were identified (Figure 4B). The spectrum of PNGase A released glycans (Figure 4C) in contrast contained only minor amounts of high mannose type, indicative of an efficient initial PNGase F digest. It was instead dominated by highly fucosylated pauci-mannose type (*m/z* 1171–2481 Fuc0-4Hex3-6GlcNAc2). MS/MS analysis of the molecular ion of m/z 1724 (Fuc2Hex4GlcNAc2) contained structurally informative fragment ions at m/z 1098, 853 and 648, which confirmed the presence of α1-3 and α1-6 fucosylation, similar to native H11 extract. These structures are shown in more detail in Additional file 3. The N-glycan profiles are fully consistent with previously published *C. elegans* profiles [18-20].

In addition, MS analysis showed evidence of phosphorylcholine (PC) modifications on rH11-4 and this was supported by reactivity of PC TEPC-15 antibody (Figure 4D). PNGase F released N-glycans were treated with HF, which cleaves potential PC modifications, prior to permethylation and MALDI-MS analysis. The HF treated N-glycans showed an increase in abundance of complex structures terminated with HexNAc residues (*m/z* 1416–22693 Fuc0-1Hex3-6GlcNAc2-5), as shown in Additional file 4, thus indicating probable PC substitution [43]. PC on some helminth proteins has been shown to interfere with activation of T cell and B cell responses [44,45]. We examined whether rH11 proteins had any inhibitory effect on activation of helminth-naive ovine peripheral blood lymphocytes using Con A as a polyclonal T cell activator; none was observed as shown in the lymphocyte proliferation assays in Additional file 5.

### Vaccine trials using *C. elegans*-expressed H11 protein

**Figure 5:**
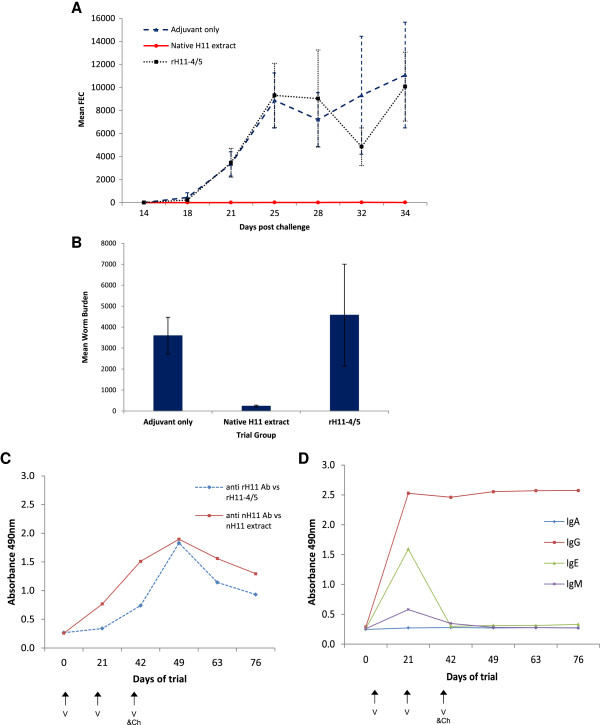
**Faecal egg output, adult worm burdens and ELISA antibody responses following vaccination with rH11-4/5 co-expressed proteins or with native H11-enriched extract.** Faecal egg count (FEC) **(A)** was monitored from days 14–34 post-challenge infection and adult worm burdens **(B)** counted at necropsy on day 35. The mean group count from lambs vaccinated with rH11-4 /5 proteins, native H11-enriched extract or Vax Saponin adjuvant are shown, with error bars representing the standard error of the mean. **(C)** ELISA OD values of antisera (1/800 dilution) reactivity to rH11-4/5 co-expressed proteins or native H11 extract following immunisation with the homologous antigen. Lambs were vaccinated on days 0, 21 and 42 and challenged with 5000 L3 on day 42, as indicated. **(D)** Ig isotype responses (1/50 dilution) to native H11-enriched extract measured by ELISA.

To examine any protection afforded by rH11 protein, six-month old lambs (7 per group) were vaccinated three times at 3 weekly intervals with rH11 or native H11-enriched extract and challenged at the time of the third vaccination. In the first trial, protection using rH11-1 and rH11-4 proteins (10 μg each protein per vaccination) was examined and a second trial tested any protection afforded by rH11-4 and rH11-5 proteins co-expressed in *C. elegans* (20 μg total protein per vaccination). In both trials, no significant difference in FEC, worm burden or worm female:male ratio at necropsy (day 35 post-challenge infection) was observed in lambs vaccinated with the *C. elegans-*expressed rH11 proteins compared to an adjuvant control group (results of trial 2 shown in Figures 5A and B and Tables 1 and 2; results of trial 1 were very similar). Lambs vaccinated with *H. contortus* native H11-enriched extract showed significant reductions in both worm burden (93.6% reduction) and FEC (99.9% reduction) compared to the adjuvant control group.

**Table 1:** Faecal egg counts (eggs per gram faeces) after challenge infection.

	**Sample**	**Days post challenge infection**						
	no.	d14	d18	d21	d24	d27	d31	d34
	1	0	183	2547	5265	4374	2808	3879
	2	0	183	3726	6057	4347	9981	9360
Adjuvant	3	0	1080	3402	9783	7794	11628	17586
control	4	0	549	4788	10998	9405	9045	15129
	5	1	51	1497	10863	5769	5400	8937
	6	0	909	3834	10791	9135	18846	13374
	7	0	183	3717	8307	9621	7551	9279
	Mean	0.17	448.3	3358.7	8866.3	7206.4	9322.7	11077.7
	SE	0.4	406.3	1053.5	2389.2	2345.0	5123.2	4594.1
	8	0	0	0	39	8	48	25
	9	0	0	0	1	0	2	4
Native	10	0	0	1	0	2	4	0
H11	11	0	0	2	11	17	0	29
Extract	12	0	0	1	4	7	4	6
	13	0	1	0	1	0	1	0
	14	0	0	0	8	20	51	26
	Mean	0	0	1	9	8	16	13
	SE	0	0	1	14	8	23	13
	15	0	480	5319	8289	7776	6435	8289
	16	0	354	2556	6966	6696	7272	9171
rH11-4/5	17	0	113	4815	12123	9504	3420	7398
	18	0	122	2412	7767	4563	3699	7884
	19	0	19	2016	5697	5562	3447	14724
	20	0	231	3771	11862	12789	3753	9054
	21	0	237	3294	12483	16335	5922	13968
	Mean	0	222.3	3454.7	9312.4	9032.1	4849.7	10069.7
	SE	0	156.7	1252.8	2782.8	4217.4	1636.5	2994.0

**Table 2:** Worm burdens at necropsy (day 35 post challenge).

	**Sample no.**	**Males**	**Females**	**Total worm count**
	1	1750	1750	3500
	2	1150	1950	3100
**Adjuvant**	3	1300	1000	2300
**control**	4	2100	1650	3750
	5	1350	2100	3450
	6	2500	2700	5200
	7	2000	1850	3850
	Mean	1735.7	1857.1	3592.9
	SE	494.7	511.1	877.2
	8	250	0	250
	9	150	100	250
**Native H11**	10	150	0	150
**extract**	11	250	0	250
	12	250	50	300
	13	150	50	200
	14	200	0	200
	Mean	200	28.6	228.6
	SE	50	39.3	48.8
	15	1150	1550	2700
	16	1200	1450	2650
**rH11-4/5**	17	3650	3400	7050
	18	4500	4250	8750
	19	1650	2750	4400
	20	900	1600	2500
	21	1800	2150	3950
	Mean	2121.4	2450.0	4571.4
	SE	1390.7	1068.9	2432.6

### Antibody response to recombinant and native H11 proteins

Characterisation of the antibody responses of lambs immunised with rH11 proteins or native H11-enriched extract were examined to identify any quantitative or qualitative differences. We focussed on serum antibody responses as previous studies have shown a correlation between protection and serum antibody level [42] and protection can be conferred by passive transfer of immune serum [42] or colostrum [46]. By ELISA, a peak in antibody response was observed 7 days after the third vaccination/challenge with native H11 extract or recombinant H11 protein (day 49; Figure 5C). Although reaching a similar maximum level, the antibody titre rose earlier (day 21) and was maintained at a higher level for longer in lambs immunised with native H11 extract. Maximum response was observed against the homologous protein used in vaccination (i.e. greater recognition of native H11 extract with antiserum from those vaccinated with this extract and vice versa). This most likely reflects the complexity of the native extract, which contains a number of other proteins in addition to H11. Antibody induced to each of these components will contribute to the ELISA response and may explain the earlier and more sustained antibody titre observed following vaccination with native H11-enriched extract.

Comparison of antibody avidity, by performing ELISAs in the presence of increasing concentrations of KSCN, showed slightly higher avidity of antibody from lambs immunised with rH11-4/rH11-5 co-expressed proteins compared to those immunised with native H11 extract (3.2 M vs 2.7 M KSCN). The antibody response to vaccination with native H11-enriched extract was predominantly of the IgG isotype, although IgE and to a lesser extent IgM responses were detected at day 21 of the trial, at the time of the second vaccination (Figure 5D). In contrast, IgG was the only isotype detected following vaccination with rH11 proteins, with no IgE or IgM response identified. Using rH11-4/rH11-5 proteins on ELISA plates, only IgG isotype was detected and, similarly, coating ELISA plates with a purer preparation of native H11 (ConA binding proteins passed over peanut lectin followed by gel filtration chromatography), no IgE nor IgM responses were detected (Additional file 6), suggesting that these may be directed to other components of the native H11-enriched extract.

**Figure 6:**
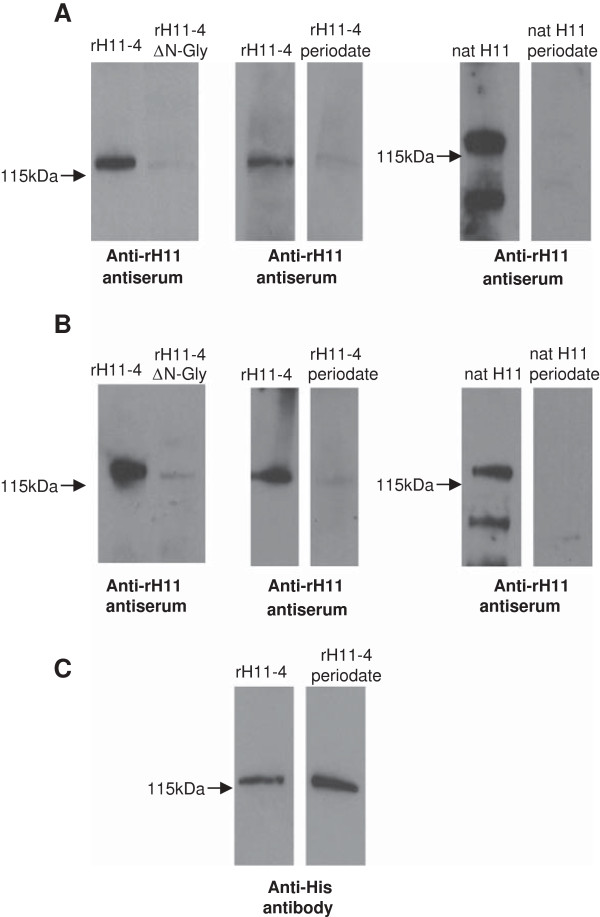
**Antibody reactivity to glycan epitopes on *C. elegans *recombinant or native H11 proteins.** Western blots showing recognition of rH11-4, rH11-4ΔN-Gly, native H11-enriched extract and native H11-enriched extract treated with mild periodate, by antibody to native H11 extract (anti-nH11; panel **A**) or to rH11-4/5 proteins (anti-rH11; panel **B**). Reactivity of anti-His antibody confirmed equal loading of rH11-4 protein (panel **C**). Proteins in addition to H11 are observed in the nH11 extract.

Antiserum from lambs vaccinated with rH11-4/5 proteins or native H11-enriched extract recognised both recombinant and native H11 proteins to a similar level by Western blot. Using this method, most of the antibody response detected was to glycan moieties on rH11, as indicated by the significantly reduced reactivity (approximately 95% reduction) to rH11-4ΔN-Gly, in which the three predicted N-glycosylation sites were mutated (Figure 6). The level of reduction was similar using antiserum to native H11 extract (Figure 6A) or rH11 protein (Figure 6B). Mild periodate treatment of rH11 or nH11 protein similarly reduced recognition by both antisera. ELISA showed a reduction in antibody reactivity to rH11-4ΔN-Gly or periodate-treated recombinant H11-4/5 or native H11 extract, but to a lesser extent than by Western blot (45-59% reduction). Therefore vaccination with rH11 proteins or native H11-enriched extract induces a significant antibody response to glycan or glycan-linked epitopes.

## Discussion

A major hurdle in the development of commercial vaccines for parasitic nematodes is the difficulty of expressing identified protective antigens in a recombinant form capable of inducing protective immunity. Currently, vaccination studies are limited by the low amounts of native protein that can be purified from parasite extracts, the requirement for parasite material from infected hosts and the cost, safety and ethical considerations of this approach. Alternative methods of producing defined sub-unit vaccines are urgently needed. In this study we examined the suitability of *C. elegans* as an alternative expression system for parasitic nematode vaccine candidates. *C. elegans* is in the same nematode clade (clade V) as many important livestock parasites and it has been shown that some of the glycosylation pathways and modifications in *C. elegans* are present in related parasitic species [18,19].

There is increasing interest in parasitic helminth glycans due to their strong antigenicity and the protective potential of some glycans [47]. For example, novel tyvelose-capped glycoproteins (TSL-1 group) are present on the larval surface of the pig nematode *Trichinella spiralis.* These are strongly antigenic and anti-TSL-1 antibodies provide protection by expelling larvae from the intestine [48]. In this study we focussed on *H. contortus* H11 aminopeptidase, which is enriched in one of the most effective vaccine preparations identified for any parasitic nematode. Native H11 has previously been demonstrated to be modified with an unusual and highly antigenic Fucα(1–3) GlcNAc moiety and we showed here by detailed MS analysis that recombinant H11 expressed in *C. elegans* is modified with very similar di and tri-fucosylated glycans to the native protein. However, the N-glycan profile is fully consistent with that of *C. elegans* and indicates some structural differences from those of native H11-enriched extract. For example, many core fucosylated glycans are additionally modified with galactose residues and no evidence could be found for fucosylated LacdiNAc (LDNF) and Galα1-3GalNAc structures. While antibody to LDNF glycan has been correlated with protection following immunisation with *H. contortus* ES antigens [49], no correlation was found between the antibody response to LDNF and protection induced by H-galGP gut complex. This could reflect differences in glycan abundance between the antigens used [50]. As shown here, much of the response to rH11 vaccination is to glycan structures, similar to the response with native H11 extract. Despite this significant anti-glycan response, no protection against *H. contortus* challenge infection was observed, suggesting that the response to glycans on H11 does not contribute to protective immunity, or that the specific protective glycan structures are not present on rH11.

Following vaccination with rH11 proteins, a high antibody titre was measured by ELISA and detected by Western blot. However this level of response was short-lived and not maintained during challenge infection. In contrast, vaccination with native extract enriched for H11 induced an earlier antibody response that was maintained at a high level during infection. In addition, IgE antibody was detected in response to vaccination with native H11-enriched extract, but not to recombinant H11 protein. While H11 is predominant in the native extract, other components have been identified by MS peptide analysis (DPK and AJD, submitted). Our findings using *C. elegans* suggest that the lack of protection observed with recombinant proteins may not be due to qualitative differences between recombinant and native proteins, such as folding as previously speculated, but due to differences in the responses induced by the different protein preparations. We suggest that the level of antibody response stimulated by the recombinant proteins may not be above a threshold level during challenge infection to provide sufficient protection. In addition, the nature of the response, including Ig isotype, may differ between the two groups and influence immune outcome. Vaccination studies using recombinant forms of the *H. contortus* H-gal-GP complex (rPEP-1 and rMEP isoforms) expressed in *E. coli* or *Pichia pastoris* also induced an antibody response of shorter duration than native H-gal-GP-enriched extract, which may also explain the lack of protection observed [51].

While the effects of additional vaccine doses and/or use of alternative adjuvants, such as iscoms or other nanoparticles, may help prolong the antibody response [52], attempting to mimic the complexity of native protein vaccines may be key to successful recombinant vaccines. Recent studies aimed at developing a recombinant vaccine against the related sheep GI nematode *T. circumcincta*, demonstrated significant reductions in FEC using a cocktail of eight recombinant proteins [53]. It is likely that multicomponent vaccines are required for adequate protection against complex metazoan parasites. Thus, combining vaccine candidate antigens into a multivalent vaccine is likely to enhance the immune response above a threshold and/or influence immune outcome.

In this study, strong antibody responses to glycan epitopes on *C. elegans* expressed rH11 and native H11 were observed. While glycans can be strongly antigenic and may induce protective responses, they can also detract from a protective immune response [44] or mimick host glycans [54], thus protecting the parasite. This is particularly relevant to carbohydrates with allergenic potential and PC modifications, which can suppress immune responses. We showed here that recombinant H11 expressed in *C. elegans* is heavily glycosylated and modified by PC. Initial findings from ovine lymphocyte activation assays using Con A indicate that rH11 has no suppressive effect on T cell activation and, while it is unclear what effect rH11 has on B cell activation, high antibody titres were generated to rH11 following immunization. The highly fucosylated glycans on H11 could serve a biological function for the parasite, such as interaction with lectins and/or ingested nutrients, rather than as decoys. *C. elegans* mutants defective in GDP-fucose biosynthesis are resistant to the effects of ingested bacterial and fungal toxins [55], suggesting that fucose-containing glycoconjugates may act as binding proteins within the nematode gut.

It is possible that the lack of protection observed with *C. elegans*-expressed rH11 proteins could indicate that H11 is not protective. However, we showed by RNA interference (RNAi) that reduction of H11 mRNA levels has a detrimental effect on worm development in vivo [56]. This suggests that knockdown of H11 affects H11 assembly and/or activity within the worm gut. Although it cannot be assumed that antibody induced by native extract has a similar effect as RNAi within the parasite, we suggest that other gut proteins in combination with H11 may be required for protection. While functional redundancy between H11 isoforms has been speculated [8], in the current study three different recombinant H11 isoforms were used in vaccination trials and antibody generated cross-reacted with all isoforms. This suggests that there is sufficient similarity for antibody to target multiple H11 proteins.

We have shown here that the *C. elegans* system is amenable to co-expression of parasite proteins and that production of sufficient protein for protection studies is feasible. Therefore further protection using multiple proteins, including other gut enzymes or ES components, co-expressed in *C. elegans* in active and post-translationally modified forms are warranted. The outcomes will be important for progressing recombinant vaccine development for *H. contortus* and other parasitic nematodes. In addition, the use of *C. elegans* glycosylation mutants or site directed mutagenesis of introduced genes can allow comparison of glycosylated and non-glycosylated forms of proteins, which is relevant to understanding induction of immunity and suppressive effects of nematode infections.

## Competing interests

The authors declare that they have no competing interests.

## Authors’ contributions

CB and DPK conceived the study; ABR carried out all *C. elegans* cloning, expression, protein and antibody characterisation; AA, SH and AD carried out the glycan analysis; AD organised and analysed the vaccine trial; TMcN contributed to ELISAs and carried out cell assays and SJL carried out the H11 scaffold and RNA seq analysis. CB drafted the paper and all authors contributed to the final draft. All authors read and approved the final manuscript.

## Supplementary Material

Additional file 1 **Expression plasmid for the *H. contortus H11-4 *gene in transgenic *C. elegans*.** Grey shading, *C. elegans cpl-1* promoter; striped shading, *H. contortus Hmcp-6* signal sequence; black shading, cDNA gene fragment encoding *H. contortus H11-4*; hatched region, *C. elegans cpl-1* 3′ UTR. A *C. elegans* synthetic intron (SI) was introduced to aid transgenic expression and a 10 amino acid His tag encoding sequence was included at the 3′ end of the cDNA to allow purification using cobalt resin. Click here for file

Additional file 2 **Amino acid sequence alignment of all five identified *H. contortus *H11 isoforms.** The transmembrane domain of each isoform (predicted using TMHMM Server v. 2.0) is indicated in bold type and the active site HELAH and GAMEN motifs shown in blue. N-glycosylation sites predicted using NetNGlyc (1.0 Server) are shown in red. Accession numbers: H11, Q10737.2; H11-1, CAB57357.1; H11-2, CAB57358.1; H11-4, CAC39009; H11-5 KF381362. Click here for file

Additional file 3 **MALDI-TOF-TOF MS/MS of PNGase A released N-glycan.** MALDI-TOF-TOF MS/MS of molecular ion detected at *m/z* 1724 (selected from spectrum shown in Figure 4C). The horizontal arrows on the spectra indicate losses from the molecular ion [M + Na]^+^ of the designated N-glycan sequences in inset. All molecular ions are [M + Na]^+^. Structures that show sugars outside of a bracket have not been unequivocally defined. Click here for file

Additional file 4 **HF treated N-linked glycans from H11-4 recombinant protein.** MALDI-TOF MS of N-linked glycans (PNGase F) from H11-4 recombinant protein before **(A)** or after HF treatment **(B)**. Profiles of N-glycans are from the 50% MeCN fraction from a C_18_ Sep-Pak (Materials and Methods). All molecular ions are [M + Na]^+^. Putative structures are based on composition, tandem MS and biosynthetic knowledge. Structures that show sugars outside of a bracket have not been unequivocally defined. Click here for file

Additional file 5 **T cell activation in the presence of rH11-1, rH11-4 and rH11-4/5.** To determine whether rH11-1, rH11-4 or rH11-4/5 had any effect on lymphocyte activation, peripheral blood mononuclear cells from a helminth-naive lamb were cultured with 5 μg/mL of the T cell mitogen Con A in the presence or absence of 1.25-5 μg/mL of rH11-1, rH11-4 or rH11-4/5. Recombinant proteins were added 30 min before Con A to limit any direct binding of the recombinant proteins to Con A. Cell proliferation was assessed by incorporation of [3H] thymidine at 72 h and was expressed as counts per minute (CPM). No significant difference in proliferation was observed between cultures stimulated with Con A alone vs. cultures stimulated with Con A + rH11-1 or rH11-4 **(A)** or Con A + rH11-4/5 **(B)**. Data represent mean of three replicates, with error bars representing the standard error of the mean. Statistical analysis was performed using Kruskal–Wallis one-way analysis of variance. Click here for file

Additional file 6 **Ig isotype responses to rH11-4/5 (A) and to a purer preparation of native H11 (B) measured by ELISA.** ELISA OD values of antisera (1/50 dilution) following immunisation with native H11-enriched extract on days 0, 21 and 42 and challenged with 5000 L3 on day 42, as indicated. Click here for file
