# Supplementary figures and images for: Novel expression of Haemonchus contortus vaccine candidate aminopeptidase H11 using the free-living nematode Caenorhabditis elegans
Source: Vet Res. 2013 Dec 1;44(1):111. doi: 10.1186/1297-9716-44-111 (PMC4176091; doi:10.1186/1297-9716-44-111)

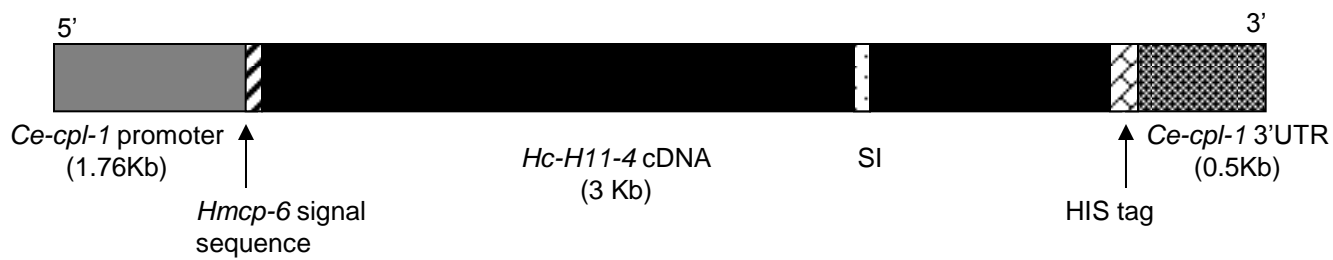

Supplement: Additional file 1 — Expression plasmid for the H. contortus H11-4 gene in transgenic C. elegans. Grey shading, C. elegans cpl-1 promoter; striped shading, H. contortus Hmcp-6 signal sequence; black shading, cDNA gene fragment encoding H. contortus H11-4; hatched region, C. elegans cpl-1 3′ UTR. A C. elegans synthetic intron (SI) was introduced to aid transgenic expression and a 10 amino acid His tag encoding sequence was included at the 3′ end of the cDNA to allow purification using cobalt resin. [file 1297-9716-44-111-S1.pdf]

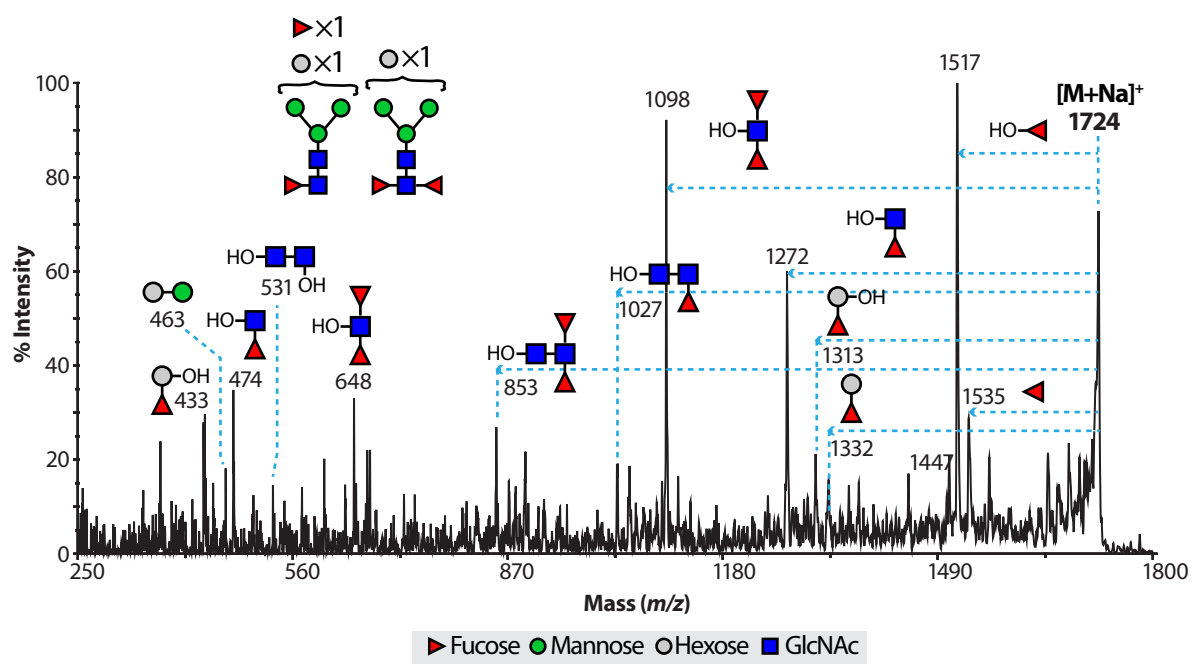

Supplement: Additional file 3 — MALDI-TOF-TOF MS/MS of PNGase A released N-glycan. MALDI-TOF-TOF MS/MS of molecular ion detected at m/z 1724 (selected from spectrum shown in Figure 4C). The horizontal arrows on the spectra indicate losses from the molecular ion [M + Na]+ of the designated N-glycan sequences in inset. All molecular ions are [M + Na]+. Structures that show sugars outside of a bracket have not been unequivocally defined. [file 1297-9716-44-111-S3.pdf]

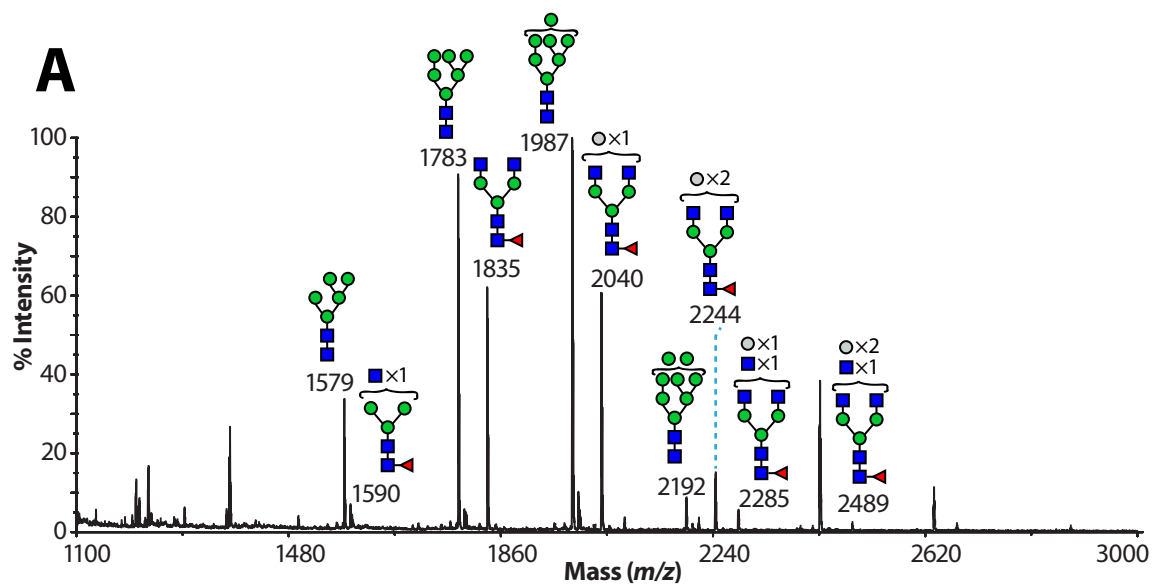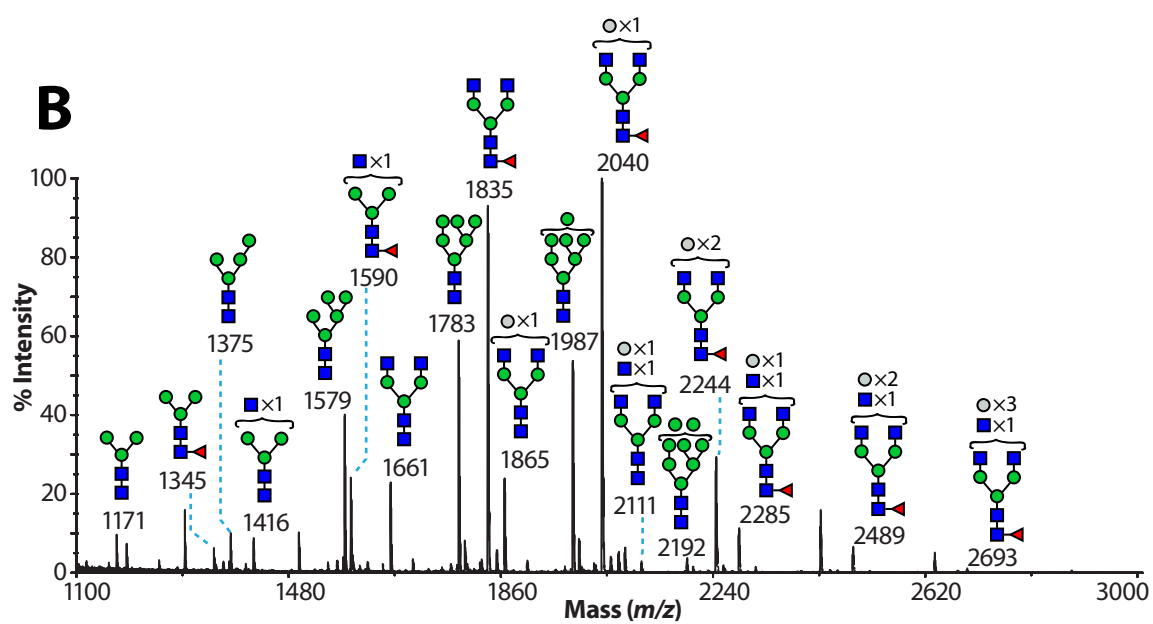

► Fucose ● Mannose ○ Hexose ■ GlcNAc

Supplement: Additional file 4 — HF treated N-linked glycans from H11-4 recombinant protein. MALDI-TOF MS of N-linked glycans (PNGase F) from H11-4 recombinant protein before (A) or after HF treatment (B). Profiles of N-glycans are from the 50% MeCN fraction from a C18 Sep-Pak (Materials and Methods). All molecular ions are [M + Na]+. Putative structures are based on composition, tandem MS and biosynthetic knowledge. Structures that show sugars outside of a bracket have not been unequivocally defined. [file 1297-9716-44-111-S4.pdf]

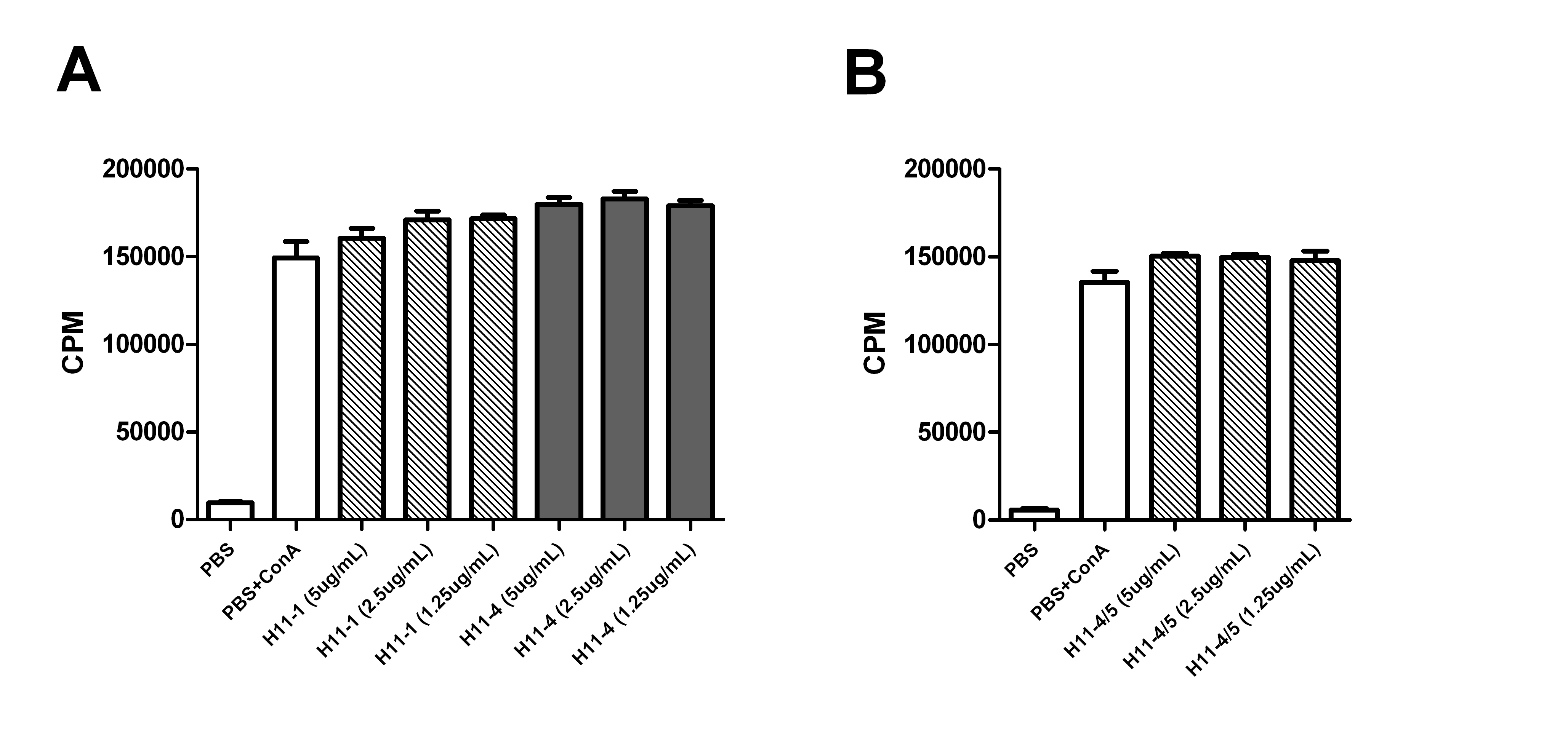

Supplement: Additional file 5 — T cell activation in the presence of rH11-1, rH11-4 and rH11-4/5. To determine whether rH11-1, rH11-4 or rH11-4/5 had any effect on lymphocyte activation, peripheral blood mononuclear cells from a helminth-naive lamb were cultured with 5 μg/mL of the T cell mitogen Con A in the presence or absence of 1.25-5 μg/mL of rH11-1, rH11-4 or rH11-4/5. Recombinant proteins were added 30 min before Con A to limit any direct binding of the recombinant proteins to Con A. Cell proliferation was assessed by incorporation of [3H] thymidine at 72 h and was expressed as counts per minute (CPM). No significant difference in proliferation was observed between cultures stimulated with Con A alone vs. cultures stimulated with Con A + rH11-1 or rH11-4 (A) or Con A + rH11-4/5 (B). Data represent mean of three replicates, with error bars representing the standard error of the mean. Statistical analysis was performed using Kruskal–Wallis one-way analysis of variance. [file 1297-9716-44-111-S5.jpeg]

**A**

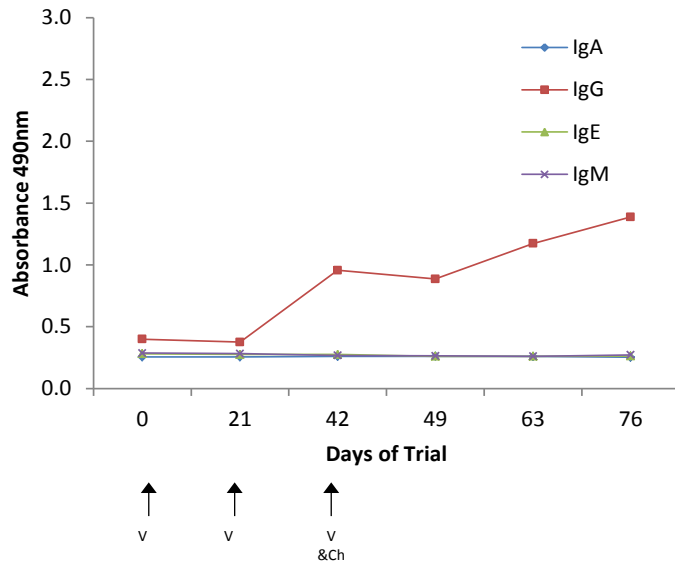

**B**

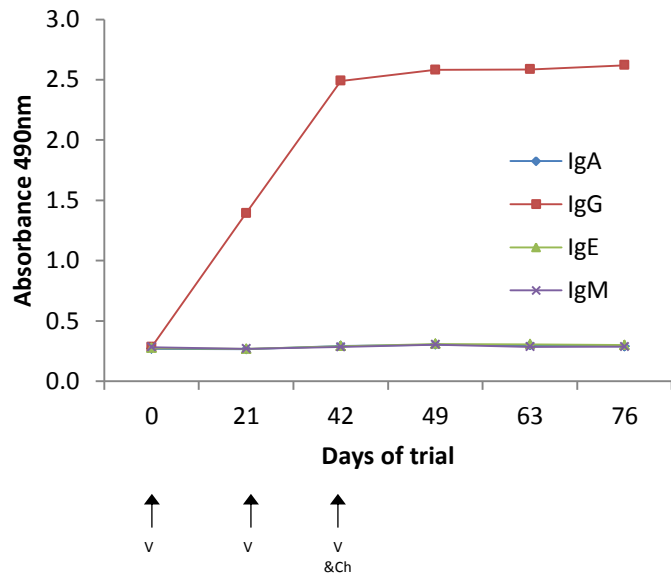

Supplement: Additional file 6 — Ig isotype responses to rH11-4/5 (A) and to a purer preparation of native H11 (B) measured by ELISA. ELISA OD values of antisera (1/50 dilution) following immunisation with native H11-enriched extract on days 0, 21 and 42 and challenged with 5000 L3 on day 42, as indicated. [file 1297-9716-44-111-S6.pdf]
