# Supplementary material for: Novel expression of Haemonchus contortus vaccine candidate aminopeptidase H11 using the free-living nematode Caenorhabditis elegans
Source: Vet Res. 2013 Dec 1;44(1):111. doi: 10.1186/1297-9716-44-111 (PMC4176091; doi:10.1186/1297-9716-44-111)
Supplement: Additional file 2 — Amino acid sequence alignment of all five identified H. contortus H11 isoforms. The transmembrane domain of each isoform (predicted using TMHMM Server v. 2.0) is indicated in bold type and the active site HELAH and GAMEN motifs shown in blue. N-glycosylation sites predicted using NetNGlyc (1.0 Server) are shown in red. Accession numbers: H11, Q10737.2; H11-1, CAB57357.1; H11-2, CAB57358.1; H11-4, CAC39009; H11-5 KF381362. [file 1297-9716-44-111-S2.pdf]

Hc H11	MTSQG---------RTRTLLNLT**PIRLIVALFLVAAAVGLSIGLTY**YFTRKAFDTSEKPGKDDTGGKDKDNSPSAAELLLPSNIKPLSYDLTIKTYLPGYVDFPPEK**NLT**
Hc H11-4	MTARE---------RKRSLVE**FTPFSLAFAMFAVAAAVGLAIGL**TYFFTRKAFDPTQKD-KDQPGGKEKDNSPSAAELLLPSNIKPLLYDLTIKTYLPGYVNFPPEK**NLT**
Hc H11-2	MTAEW---------QKRRILG**FSPISLLCTLFVLAAAVGLSIGL**TYYFTRKAFDTSQKEQKDDTGGKEKDNSPSAEELLLPTNIKPVSYDLSIKTYLPGYVNFPPEK**NLT**
Hc H11-1	MTAEESQEQETQQPRKNTVLRLTPIK**SLFALLVVAAAVGLSIGLTYYFT**RKAFDTTGGNGK-EDQPIVDDNSPSAEELRLPTTIKPLTYDLVIKTYLPNYVNYPPEKDFA
Hc H11-5	MTVQW---------TKRTVLKFT**PITLLLLLFLVAASIGLSIGLTY**YFTRKAYDTTEKN---K-DHGADDNSPSAEELRLPKNIEPLLYDLSIKTYLPGYVSFPPEK**NLT**
Hc H11	FDGRVEISMVVIEPTKSIVLNSKKISVIPQECELVSGDKKLEIESVKEHPRLEKVEFLIKSQLEKDQQILLKVGYIGLISNSFGGIYQTTYTTPDGTPK-IAAVSQNEPI
Hc H11-4	FDGRVEISMVVVEPTKSIVLNAKKITVIPAECEVLSGTQKLDIESVKEHERLEKLEFRLKSRLEKDQKILLKITYAGLISNTLGGIYQTTYTDANGNPK-IAAVSQNEPI
Hc H11-2	FDAHVEISMVVVEPTNSIVLNSKKITLAQGGCELFSGNQKLDIESVKMQERLDKLEITLKNQLQKEQKILLKITYTGLISNTLGGLYQSIYTDTDGKTK-IVAVSQNEPS
Hc H11-1	IDGTVVIAMEVVEPTKSIVLNSK**NIS**VIADQCELFSNNQKLDIEKIVDQPRLEKVEFVLKKKLEKNQKITLKIVYIGLINDMLGGLYRTTYTDKDGTTK-IAACTHMEPT
Hc H11-5	FDGQVGISLRVVEPTKSIVLNSK**NIT**VIPDKCELFSGDKKLEIESIKEHERLEKLEILLKNRLEKDQEVLLKVGYTGIISNTLGGLYQATYTDTDGTVKRIAAATQHCPS
Hc H11	DARRMVPCMDEPKYKA**NWT**VTVIHPKGTKAVSNGIEVNGDGEISGDWITSKFLTTPRMSSYLLAVMVSEFEYIEGETKTGVRFRIWSRPEAKKMTQYALQSGIKCIEFYE
Hc H11-4	DGRRMVPCMDEPKYKA**NWT**VTVIHPKGTKAASNSIEINGEGDVSGDWITSKFETTPRMSSYLLAVFISEFDFVEGRTKQDVRFRIWSRPEAKGMTKYALESGIKCIEFYE
Hc H11-2	DARRIAPCFDEPKYKAKWTVTVVHPKGTKAASNGIEANGNGELQGDWITSKFKTTPPMSSYLLAIIVCEFEYIEGKTETGVRFRIWSRPEAKAMTAYALDAGIRCLEFYE
Hc H11-1	DARLMVPCFDEPTFKA**NWT**VTVIHPKGTSAVSNGIEK-GEGEVSGDWVTTRFDPTPRMSSYLIALVISEFKYIE**NYT**KSGVRFRIWARPEAMKMTEYAMIAGIKCLDYYE
Hc H11-5	DARRLVPCLDEPSFKASWTVTVIHPKGTKAVSNGIETNGKGEVSGDWIISKFETTPRMSSYLLAVVVSEFDYIEGFTKSGVRFRIWSRPEAK**NMT**AYARDAGIRCLEFYE
Hc H11	DFFDIRFPLKKQDMIALPDFSA**GAMEN**WGLITYRENSLLYDDRFYAPMNK--QRIARIVA**HELAH**QWFGDLVTMKWWDNLWLNEGFARFTEFIGAGQITQDDARMRNYFL
Hc H11-4	DFFDIKFPLKKQDMIALPDFSA**GAMEN**WGLITYRENSLLYDEKFYGPTNK--RRVAVVVA**HELAH**QWFGDLVTMKWWDDLWLNEGFATFVEYIGADQIGDHYFNMPDFFL
Hc H11-2	KFFDIKFPLEKQDMIALPDFTA**GAMEN**WGLITYREDSLLYDEKIYAPMNK--QRVALVVA**HELAH**QWFGNLVTLKWWDDTWLNEGFATFVEYLGMDEISHNNFRTQDFFL
Hc H11-1	DFFGIKFPLPKQDMVALPDFSS**GAMEN**WGLITYREGSVLYDENLYGPMNK--ERVAEVIA**HELAH**QWFGNLVTMKWWDNLWLNEGFASFVEYIGADFISDGLWEMKDFFL
Hc H11-5	NFFDIKFPLKKQDMVALPDFSF**GAMEN**WGLITYRESSLLYDDRYYTPIIQATQLVALVVA**HELAH**QWFGDLVTLKWWDDLWLNEGFARFVEYIGTDEIN**NKT**IRMDDFFL
Hc H11	IDVLERALKADSVASSHPLSFRIDKAAEVEEAFDDITYAKGASVLTMLRALIGEEKHKHAVSQYLKKFSYSNAEATDLWAVFDEVVTDVEGPDGKPMKTTEFASQWTT--
Hc H11-4	IGALERALKADSAASSHPLSFRIDKAVEVEEAFDDISYAKGASIITMLRALIGEDKHKHAVTQYLKKFSYSNAQASDLWEVFDEVVTDIRGPDGKPMKTTAFADQWTT--
Hc H11-2	LDGMDRGMRADSAASSHPLSFRIDKAAEVAEAFDDISYAKGASVLTMLRALIGEDNYRNAVVQYLKKFSYSNAQAADLWNVFNEVVKGVKGPDGNVMKIDQFTDQWTY--
Hc H11-1	LAPYTSGITADAVASSHPLSFRIDKAADVSEAFDDITYRKGASVLQMLLNLVGDENFKQSVSRYLKKFSYDNAAAEDLWAAFDETVQGITGPNGGPLKMSEFAPQWTT--
Hc H11-5	PNVLVKALDADAVSSTHPLSFRVDKAAEVVEAFDRITYEKGASVLKMLQALIGQKNYKQAVTQYLRKFSYSNAQASDLWDVFDEVVKDVKGPDGNLMKTTEFASQWTTQV
Hc H11	QMGFPVISVAEFNSTTLKLTQSRYEANKDAVEKEKYRHPKYGFKWDIPLWYQEGDKKEIKRTWLRRDEPLYLHVSDAGAPFVVNADRYGFYRQNHDANGWKKIIKQLKDN
Hc H11-4	QMGFPLVTVEAF**NAT**SVKISQSRFKTNKDAKEPEKYRHPKYGFKWDIPLWYQEGDNKEVKQTWIRREEPLYLHVNDLSKPFVVNADRHGFYRQNYDADGWRKIIKQLRDN
Hc H11-2	QMGYPVVKVEEFNATSLKVTQSRYKTNKDALEPEKYRNPKYGFKWDVPLWYQEGNSKEVKRTWLKRDEPLYLNVNNRDISLVVNADRHGFYRQNYDANGWKKIINQLKKD
Hc H11-1	QMGFPVLTVESVNATTLKVTQKRYRQNKDAKEPEKYRHPTYGFKWDVPLWYQE-DEQQVKRTWLKREEPLYFHVSNSDSSVVVNAERRAFCRSNYDANGWRNIMRRLKQN
Hc H11-5	QMGFPLVTVKAF**NAT**SLQITQTRYKTNKDALEPEKYRHPKYGFKWDVPLWYQEGDNKDIKFAWLTREKPLYLHMTKPDTTIVVNADRHGFYRQNYDANGWRKIIKQLKKN
Hc H11	HEVYSPRTRNAIISDAFAAAATDAIEYETVFELLNYAEKETEYLPLEIAMSGISSILKYFGTEPEAKPAQTYMMNILKPMYEKSSIDFIANNYRNDKLFFQINLQKDVID
Hc H11-4	HKVFSPRTRNAIISDAFALASVNAIEYETVFELLKYAVNEEEFIPWTEAISGIFAVLKFFGNEPESKPAEAYMMKILEPMYKKSDLGYIAAKYKDDQLFSKINLQKDIID
Hc H11-2	HKVFGPRTRNAIISDAFAAATIDAIDYETVFELLEYAKNEEEFLPWKEALSGMFAVLKFFGNEPETKPARAYMMSILEPMY**NKS**SIDYIVKNYLDDTLFTKINTQKDIID
Hc H11-1	YKVYGPRTRNALISDAFAAAAVEEMDYETVFEMLKYTVNEEDYLPWKEAISGFNTILDFFGSEPESQWASEYMRKLMKPIYDKSSIKFIAENYKKDKLFFKNNLQIAVIS
Hc H11-5	HKVCSARTRNAIIGDAFAAALIDELEYETVFKLLEYAKNEEEYLPWTEAISGFYAILDFFGNEPESISAKAFMKNILKPMYKKTSMKYIADNYENDSLFFEVNLQTSIID
Hc H11	MFCALGSQDCRKKYKKLFDDEVMNKCRDGQAATECVRIAAPLRSSVYCYGVKEGGDYASDKVMELYTAETLALEKDFLRLALGCHKDVTALKGLLLRALDRNSSFVRMQD
Hc H11-4	AYCALGSKDCMKKYKDIFDREVMNKCNDGDEATKCVSVAAPLRSSTYCNGVKAGGTYAFEKVKALYYEETVQLEKDMLLRALGCHRDVTALKGLLLLAVDRNSSFVRLQD
Hc H11-2	AYCSLGSKDCIKQYKDIFYDEVMPKCKAGEAATKCVKVSAPLRANVYCYGVQEGGEEAFEKVMGLYLAEDVQLEKGILFKALACHKDVTALKELLLRALDRKSSFVRLQD
Hc H11-1	TYCALGGKECLEEMKKLFDEEVMKKCRPGQQATDCVKVTAPLRKTVYCYGVQEGGDEAFDKVMELYNAEQVQLEKDSLREALGCHKDVTALKGLLMLALDRNSSFVRLQD
Hc H11-5	AYCFLGARECIKNYADLFDKEVMKKCKDGDKASKCVSIAAPLRAKAYCYGVKEGGEAAFDKVMKLYYAENVQLEKDVLLQGLGCHKDITALKRLLLLALDRNSSFVRLQD
Hc H11	IPSAFNDVAANPIGGEFIFNFLIERWPDIIESIGTKHTYVEKVIPACTSGIRSQQQIDQLKNLQKNGMNARQFGAFDKAIERAQNRVDWIKKHFQKLAAFFKKATL-
Hc H11-4	IPNAFQAVAANPVGEEFMFNFLIERWGDIIGSIGSEPTYVERVIPPCTSGIRSKQQIDQLRNLHKNGIHAQEYSTFVKEIERAEHKVDWIKKHFKKLASFFKNATW-
Hc H11-2	VPTAFRAVSENPVGEEFMFNFLMERWEEITASLETEHRAVDKVVGACCTGIRSQQQIDQLKNLQKNNAQAKKFGSFTQEIEKGEHKIAWIKKHFHRLSEFFKRARS-
Hc H11-1	AHDVFNIVSRNPVGNELLFNFLTERWEEILESLSIRHRSVDRVIKACTRGLRSREQVQQLKNLYKNDKRAREYGAFGGAIERSEHRVKWIEKHFRKLAAFFKKSNS-
Hc H11-5	VAAVYYAVSSNPIGKEFMFNFLLERWEEILEGLTTEHRAVERVIKACTAGIRLEQQIDQLRSLQKNGEHAREYGAFDGQIERAEHKINWIKKHMRKLSDFFEKSTR-
